# Diacylglycerol Kinases Balance DAG Signaling to Control B Cell Maturation, Germinal Center Homeostasis, and Humoral Immunity

**DOI:** 10.64898/2026.10.05.756823

**Published:** 2026-10-12

**Authors:** Jinhai Xie, Shimeng Zhang, Wendy Lee, Huishan Tao, Yun Pan, Xunrong Luo, Xiao-Ping Zhong

## Abstract

Diacylglycerol (DAG) is a critical second messenger downstream of the B-cell receptor (BCR), but how DAG signaling is regulated during B-cell development and humoral responses remains incompletely understood. Here, complementary gain- and loss-of-function approaches revealed stage-specific requirements for diacylglycerol kinase (DGK)-regulated signaling in B cells. Expression of the gain-of-function DGKζ mutant DGKζΔNLS had limited effects on early BM B-cell development but progressively impaired transitional and mature B-cell populations. DGKζΔNLS attenuated BCR-induced ERK1/2 and mTOR signaling, activation, and proliferation and, when expressed in AID-expressing B cells, reduced GC B-cell survival and T-dependent IgG responses without detectably impairing GC B-cell proliferation in vivo. Conversely, combined DGKα/ζ deficiency resulted in marked spontaneous GC B-cell expansion associated with enhanced ERK1/2, Akt, and S6 signaling and increased glucose uptake. Despite this steady-state GC expansion, DGKα/ζ-deficient mice developed spontaneous autoreactivity and mounted impaired antigen-specific antibody responses. Thus, B-cell developmental stages exhibit differential sensitivity to altered DGK activity, and opposing perturbations of DGK-regulated signaling produce reciprocal effects on GC B-cell accumulation while both compromise productive humoral immunity. These findings identify DGKs as critical regulators that tune lipid signaling for B-cell maturation, GC homeostasis, and effective antibody responses.

## Full Text Availability

The license terms selected by the author(s) for this preprint version do not permit archiving in PMC. The full text is available from the preprint server.

